# Vitamin D Receptor Gene Polymorphisms and Their Association with Antenatal Depression: A Prospective Cohort Study in Malaysia

**DOI:** 10.1192/j.eurpsy.2026.10190

**Published:** 2026-07-31

**Authors:** N. Salibie

**Affiliations:** Universiti Sains Malaysia, Penang, Malaysia

## Abstract

**Introduction:**

Antenatal depression (AND) is considered one of the most prevalent medical complications during pregnancy. In Malaysia, the prevalence of AND varies across different regions, ranging from 8.4% to 20.3% in recent studies [1,2]. Genetic variations in the vitamin D receptor *(VDR)* gene have been associated with the development of depressive symptoms [3,4]. However, its relationship with AND is yet to be explored.

**Objectives:**

This study aimed to investigate the prevalence of AND and the role of genetic variations in the *VDR* gene in the development of this disorder

**Methods:**

This is a prospective cohort study that includes subjects who were recruited in their 36 – 38 weeks of gestation and attending the Obstetrics & Gynecology Clinic, Hospital Pakar Universiti Sains Malaysia (HPUSM). VDR polymorphisms (ApaI, TaqI, FokI, and BsmI) were genotyped using the Amplification Refractory Mutation System (ARMS-PCR) technique.AND was determined using the Malay version of the Edinburgh Postnatal Depression Scale (EPDS) with a cut-off point of 12. Statistical analysis for risk factors of AND was determined using the Chi-square test and logistic regression in IBM SPSS Statistics version 27.0.

**Results:**

A total of 207 subjects were recruited in this study, with mean years of 33 (IQR**:** 29–36 years)**.** The prevalence of AND symptoms is 7.5% (n=15). Results showed that subjects who felt anxious during their current pregnancy have nine times higher odds of experiencing AND symptoms compared to those who did not have anxiety ( OR = 9.56, 95% CI: 2.92 - 31.32, P= < 0.001). A higher percentage of subjects with VDR-ApaI allelic variant (A allele) were found to have AND according to the Chi-square test (P= 0.043), while further analysis using the logistic regression test showed a trend of increased risk, although not significant (OR = 4.27, 95% CI: 0.93 - 19.5, P= 0.061). On the other hand, our results found that there is no association between the presence of each of VDR-SNPs (TaqI, BsmI and FokI) and AND among subjects.

**Conclusions:**

The prevalence of AND among our study population is 7.5%. Our findings suggest that ApaI could be considered a promising marker for AND development, which requires further investigation in a larger population.

References:

Arifin et al. IIUM Med J Malaysia 2021; 20(1).

Narayanan et al. Front Psychiatry 2024; 15:1466074

Glocke et al. Kidney Blood Press Res 2013; 37:311–22.

da Silva Sabião et al. Sci Rep 2024; 14:1–11.

**Disclosure of Interest:**

None Declared

